# Multiple basal infusion rates in open-loop insulin delivery systems: is there a metabolic benefit?

**DOI:** 10.20945/2359-4292-2023-0055

**Published:** 2024-02-23

**Authors:** Juliana Marques Sá, Sara de Campos Lopes, Maria Joana Santos, Marta Alves, Adriana De Sousa Lages

**Affiliations:** 1 Serviço de Endocrinologia do Hospital de Braga Braga Portugal Serviço de Endocrinologia do Hospital de Braga, Braga, Portugal

**Keywords:** Type 1 diabetes, diabetes mellitus, hypoglycemia, insulin pumps, continuous subcutaneous insulin infusion

## Abstract

**Objective:**

To evaluate glycemic control according to the number of daily basal rates (BRs) in type 1 diabetes patients using continuous subcutaneous insulin infusion (CSII).

**Subjects and methods:**

Cross-sectional study of patients treated with an open-loop CSII for at least 6 months and using a flash glucose monitoring system. Patients were divided into 2 groups: group 1 (G1) and group 2 (G2), with ≤4 and >4 BRs/24h, respectively. The groups were compared regarding HbA1c, time in range (TIR), time above range (TAR), time below range (TBR), glucose management indicator (GMI), glucose variability and data related to hypoglycemia. Regression models were performed.

**Results:**

The study included 99 patients (n = 55 in G1; n = 44 in G2). Median (Interquartile range) overall age was 30 (17) years, with 19.5 (48) and 51 (77) months of CSII use, respectively. The median number of different BRs was 3 (2) for G1 and 6 (2) for G2. There were no differences concerning age, sex, educational stage, weight, and insulin analog used. G2 had longer disease duration, longer CSII use, and higher total basal daily dose/kg. No significant differences regarding HbA1c, median glucose, GMI, TIR, TAR, and CV were found. G2 patients had more hypoglycemia, more asymptomatic hypoglycemia, and higher TBR. After adjusting for potential confounders, G1 maintained a lower risk of asymptomatic hypoglycemia.

**Conclusion:**

Programming open-loop CSII devices with more than 4 BRs does not improve metabolic control. Additionally, it seems to be a risk factor for hypoglycemia and was an independent predictor for asymptomatic hypoglycemia.

## INTRODUCTION

Type 1 diabetes mellitus (T1D) results from autoimmune destruction of pancreatic beta cells, leading to a total or nearly total insulin deficiency (1,2). Treatment of T1D patients aims to mimic the physiological glycemic pattern seen in nondiabetic individuals through intensive insulin therapy regimens, which include multiple daily insulin (MDI) administrations or continuous subcutaneous insulin infusion (CSII) systems (1,2).

The use of CSII has gradually acquired greater expression as the most physiological method for insulin delivery. This system replicates a healthy individual’s pancreatic insulin secretion by releasing relatively small amounts of insulin during fasting – balancing hepatic gluconeogenesis – and by administering prandial insulin bolus to cover the glycemic excursion after meals containing carbohydrates (3-6).

In contrast to the MDI therapeutic strategy, CSII systems have the possibility to adjust the amount of insulin infused per hour (basal rate [BR]), allowing for distinct and individualized hourly rates. If properly programmed, this system is associated with significant reductions in hemoglobin A1c (HbA1c) without increasing severe hypoglycemia frequency (7-9).

In healthy people and T1D individuals, basal insulin requirements vary throughout the day based on the circadian rhythm (4,10-14). The greatest demand occurs in the morning, from 4:00 to 8:00 AM, and is related to the secretion of insulin-antagonistic effect hormones, such as glucagon, adrenaline, and cortisol. During this period, there is a spontaneous increase in plasma glucose, the so-called dawn phenomenon (12,14,15). Insulin requirements decrease throughout the day, reaching their nadir at the beginning of the night (13,14). Based on these data regarding the physiological insulin pattern throughout the 24-hour day, it is common to program the CSII initially with 2 to 4 insulin BRs to meet an individual’s various requirements (4,13,16).

Bachran and cols. (17) demonstrated that patient’s age is the main determinant of total daily insulin requirements and of the circadian distribution of insulin BRs. In line with this, Scheiner and Boyer showed that basal insulin requirements in T1D patients are not adequately met with a single 24-hour basal infusion rate. They stated that in adults over 20 years of age, insulin requirements increase abruptly in the early morning hours, fall until midday, remain low and stable throughout the afternoon, and gradually increase in the early night (6). However, Tildesley and cols. refuted these data, concluding that a single BR was not significantly inferior for glycemic control in T1D with respect to HbA1c value (8).

To date, there is scarce evidence regarding the impact of multiple insulin BRs on T1D patients’ glycemic control, especially if the latest recommended metrics, namely time in range (TIR), time above range (TAR), time below range (TBR), and glycemic variability, are considered. The aim of this study was to assess and compare glycemic control using these new metrics and to evaluate the frequency of acute diabetes-related complications according to the number of BRs in T1D patients treated with CSII.

## SUBJECTS AND METHODS

### Study design and study population

The present study was a retrospective cross-sectional study that included adult T1D patients treated with CSII followed up with in our Endocrinology Department at Hospital de Braga using an interstitial glucose monitoring system concomitantly.

Exclusion criteria considered in our sample were age under 18, pregnancy, usage of hypoglycemic drugs other than insulin, use of the interstitial glucose monitoring system for less than 3 months prior to the date of the visit when the data were collected, less than 90% active sensor time, treatment with CSII for less than 6 months, and changes in total daily insulin dose of more than 10% in the previous 3 months (from the date of the visit when the data were collected).

**Figure 1:**
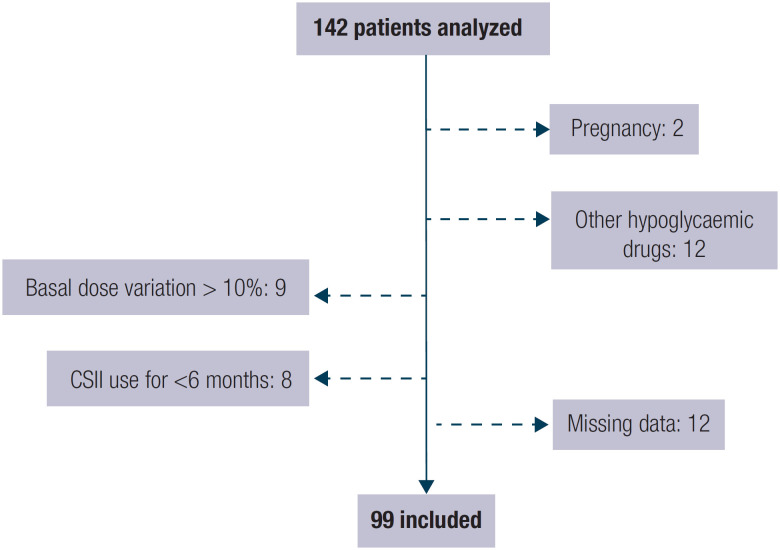
Flowchart of the study population and sample size for the final analysis.

Patients whose clinical records were unreliable or largely incomplete were also excluded. From an initial selection of 142 patients, a total of 99 patients were included in our sample (Figure 1).

Data were collected from the patients’ electronic medical records, specifically from the most recent routine clinic visit, and included demographic, anthropometric, and clinical variables as well as the latest HbA1c measurement.

We defined the use of 4 BRs as the cutoff value because it is the median number of BRs in our sample; moreover, it coincided with the usual 2 to 4 BRs recommended when one starts therapy with CSII (4,13,16). Therefore, group 1 (G1) included patients with ≤4 BRs/24 hours and group 2 (G2) included patients with >4 BRs/24 hours.

To assess glycemic control, the most recent available HbA1c and the interstitial glucose monitoring system report from the previous 4 weeks in relation to the consultation were collected, with a target range of 70-180 mg/dL. The TIR (defined as the percentage of time with glucose values between 70 and 180 mg/dL), TAR (defined as the percentage of time with glucose values greater than 180 mg/dL), and TBR (defined as the percentage of time with glucose values lower than 70 mg/dL), glucose management indicator (GMI), and glucose variability were recorded.

To assess the frequency of diabetes-related acute complications and their treatment, the number of hypoglycemia events (number of times the glucose value was less than 70 mg/dL) was retrieved from the aforementioned report, and the presence of asymptomatic hypoglycemia and the number of episodes of diabetic ketoacidosis (DKA) in the previous year were obtained by consulting the patients’ clinical records. Asymptomatic hypoglycemia was defined as a confirmed capillary blood glucose level below 70 mg/dL without typical symptoms or signs of hypoglycemia.

All participants’ confidentiality and anonymity were ensured. The study was conducted with the approval of the local ethics committee (ethics committee identifier: 156_2021).

### Statistical analysis

Statistical analysis was conducted using the IBM Statistical Package for the Social Sciences^®^ (SPSS), version 27.0 (IBM Corp., Armonk, NY, USA). Categorical variables were described as absolute and relative frequencies. For continuous variables, normality was assessed using the Shapiro-Wilk test and evaluation of histograms and was verified with the assessment of asymmetry and kurtosis.

The continuous variables for which the normality assumption was satisfied were described as mean and standard deviation. The continuous variables for which normality assumption was not satisfied were described as the median and interquartile range (IQR).

The chi-square test and Fisher’s exact test were used to assess associations between categorical variables. To compare continuous variables between two independent groups, the *t* test for independent samples (t) or the Mann-Whitney test (U) was applied, depending on the variable’s normality.

A 95% confidence interval was used, and statistically significant results were considered for *p* values below .05.

Binary logistic regression models were used to understand more clearly the impact of the number of insulin BRs on glycemic control and frequency of diabetes-related acute complications.

## RESULTS

Table 1 summarizes the characteristics of our sample according to groups.

**Table 1:** Baseline characteristics of group 1 (≤4 BR/24h) and group 2 (>4 BR/24h)

		Group 1 ≤4 BR/24h	Group 2 >4 BR/24h	P
		n=55 (55.6%)	n=44 (44.4%)	
**Sociodemographic data**				
Age (years)*		31 (18)	28 (17)	.743
Gender				
	Male	25 (45.5%)	19 (43.2%)	.842
	Female	30 (54.5%)	25 (56.8%)	
Age at T1D diagnosis (years)*		17 (14)	11 (7)	**.001**
Diabetes duration (years)*		12 (14)	14.5 (12)	**.016**
Scholarity				
	Bachelor’s or superior	19 (45.2%)	12 (41.4%)	.951
	Attending university	15 (35.7%)	10 (34.5%)	
	High school	7 (16.7%)	6 (20.7%)	
	Basic education	1 (2.4%)	1 (3.4%)	
Weight (kg)*		65 (14.7)	71 (16)	.261
**Treatment data**	
CSII Model				
	Accu-Chek^®^ Aviva/Spirit Combo	31 (56.4%)	24 (54.5%)	.746
	MiniMed^®^ Veo™ Paradigm™ System	24 (43.6%)	19 (43.2%)	
	MiniMed™ 640G	-	1 (2.3%)	
CSII use time (months)*		19.5 (48)	51 (77)	**<.001**
Rapid-acting insulin analogs				
	Fiasp^®^	28 (50.9%)	22 (50%)	.508
	Humalog^®^	8 (14.5%)	11 (25%)	
	Lyumjev^®^	9 (16.4%)	3 (6.8%)	
	Apidra^®^	6 (10.9%)	4 (9.1%)	
	Novorapid^®^	4 (7.3%)	4 (9.1%)	
Ultra-fast acting insulin analogues				
	Yes	37 (67.3%)	25 (56.8%)	.304
	No	18 (32.7%)	19 (43.2%)	
Total insulin daily dose (U)*		38.8 (15.7)	44 (24.9)	.191
Total insulin daily dose (U/kg)*		0.59 (0.25)	0.63 (0.26)	.412
Basal daily dose (U)*		16.7 (7.75)	21.78 (13.10)	**.009**
Basal insulin daily dose (U/kg)†		0.25 ± 0.08	0.31 ± 0.10	**.006**
Bolus insulin daily dose (U/kg)†		0.38 ± 0.16	0.36 ± 0.14	.419
Number of BR*		3 (2)	6 (2)	**<.001**
Time of the longest BR interval (hours)*		12 (7)	8 (3)	**<.001**
Time of the shortest BR interval (hours)*		3 (1)	2 (1)	**<.001**
Higher infusion rate*		0.80 (0.35)	1.20 (0.55)	**<.001**
Lower infusion rate*		0.65 (0.35)	0.65 (0.52)	.386
Variability of infusion rate*		0.15 (0.17)	0.40 (0.25)	**<.001**
Glycemic control data				
Last HbA1c (%)†		7.3 ± 0.9	7.3 ± 0.8	.771
Average glucose (mg/dL)†		159.9 ± 25.8	154.3 ± 24.6	.356
GMI (%)†		7.2 ± 0.8	7.1 ± 0.9	.799
TIR (%)†		66.6 ± 15.8	59.8 ± 13.8	.789
TAR (%)†		33.2 ± 16.5	32.5 ± 15.9	.825
TBR (%)*		4 (5)	6 (9)	**.049**
Glucose Variability (%)†		36.6 ± 8.0	42.2 (9.2)	.157
Diabetes-related acute complications data	
Number of DKA episodes in the last year		0	0	-
Occurrence of hypoglycemia				
	Yes	10 (21.3%)	18 (47.4%)	**.019**
	No	37 (78.7%)	20 (52.6%)	
Occurrence of asymptomatic hypoglycemia				
	Yes	1 (2.1%)	6 (16.7%)	**.040**
	No	45 (97.8%)	30 (83.3%)	

* Median (IQR).

† Mean ± SD.

BR: basal rates; CSII: continuous subcutaneous insulin infusion; DKA: diabetic ketoacidosis; GMI: glucose management indicator; TAR: time above range; TBR: time below range; TIR: time in range; T1D: type 1 diabetes.

We regarded the variability of the number of BRs as the difference between the maximal and minimal BR per hour, and it does not take into consideration the number of BR intervals. Data regarding glucose variability was only available in 28 patients’ electronic medical records.

Regarding the comparison between G1 and G2 (Table 1), G2 patients were younger at T1D diagnosis and had a longer disease duration. There were no other differences in the sociodemographic data.

*All patients were using the interstitial flash glucose monitoring system FreeStyle®* Libre. Regarding the CSII models, the vast majority (99%) were using the *Accu-Chek® Aviva/Spirit Combo and MiniMed® Veo™ Paradigm™ System. Only one patient was using MiniMed™ 640G*, but due to cost limitations, he was unable to purchase the continuous glucose monitor sensors and was also using the FreeStyle^®^ Libre.

G2 patients had been using CSII systems for a longer time and had larger total daily basal insulin doses (units per day) than G1 patients: 21.78 (13.10) *vs.* 16.7 (7.75), *P* = .009 (Md [IQR]). After adjustment for weight, G2 patients maintained a higher total basal insulin daily dose (units per kg per day). As expected, G2 patients had more BRs and hence fewer hours in each BR interval. They also presented a higher infusion rate (larger amount of insulin perfused per hour) and a greater variability of infusion rates than in G1. There were no statistically significant differences between groups regarding remaining therapeutic data, namely CSII model, type of insulin analog used, and total daily dose of insulin (basal and bolus).

In relation to glycemic control, there were no differences in HbA1c, mean glucose, GMI, TIR, TAR, or glucose variability. However, G2 patients presented a higher TBR.

Regarding acute diabetes-related complications, G2 patients showed a higher rate of hypoglycemia and asymptomatic hypoglycemia.

To characterize the differences found between groups in terms of hypoglycemia and asymptomatic hypoglycemia rates more accurately, we used binary logistic regression models to clarify whether the number of BRs was an independent predictor of these outcomes. We adjusted the model for possible confounding factors, namely disease duration, time in CSII therapy, variability of infusion rate, and basal insulin daily dose (U/kg). We established no independent predictors of hypoglycemia. We found that using 4 or less BR (i.e., G1) was associated with a lower risk of asymptomatic hypoglycemia (OR 0.06, 95% CI, 0.004-0.819; *P* = .035).

## DISCUSSION

Our study shows that programming open-loop CSII devices with more than 4 BRs does not significantly improve metabolic control. Additionally, it seems to be a risk factor for hypoglycemia and was an independent predictor of asymptomatic hypoglycemia. We found no differences between groups with different numbers of BRs regarding more recent and more relevant glucose metrics obtained through the use of flash glucose monitoring systems, namely the average glucose, GMI, TIR, TAR, and glucose variability. To the best of our knowledge, this was the first study taking into consideration these newer metrics when assessing possible differences regarding the number of BRs used. These metrics allow for a more complete picture of the daily glucose variations and patterns, compared to HbA1c alone (18).

Published data show that it is common to program CSII systems initially with 2 to 4 BRs to account for the individual’s distinct physiological insulin requirements throughout the day (4,13,16). The median number of BRs in our sample was 4, so half of our patients had their CSII programmed with ≤4 BRs and the other half had more BRs defined than is theoretically explained by physiological insulin requirements.

Researchers have conducted few studies to evaluate the impact of the number of BRs on glycemic control in T1D patients. Most of them have been conducted with children and adolescents, and the results are contradictory. Nabhan and cols. (19) found that children and adolescents with good metabolic control, evaluated by HbA1c, had an average of 4.4 ± 1.3 BRs (*vs.* 3.4 ± 1.1 BRs in patients with poor glycemic control) and concluded that more BRs was predictive of better diabetes control. These authors stated that the number of BRs may serve as a surrogate for the intensity and frequency of insulin adjustment. Nonetheless, McVean and cols. (20), who also studied children and adolescents, found no differences in the numbers of BRs based on metabolic control (also evaluated exclusively using HbA1c).

Regarding T1D adult patients, in 2005, Scheiner and Boyer (6) showed that basal insulin needs are not adequately met with a flat rate of insulin delivery for 24 hours, and more than 85% of the participants in their study demonstrated distinctive "peaks" and "drop-offs" in basal insulin requirements at some point during the day. In a study published in 2012 aiming to determine whether there are differences regarding HbA1c levels between T1D adult patients utilizing different numbers of BRs, Tildesley and cols. (8) concluded that a single BR was not significantly inferior in glycemic control. In line with these studies but with different cutoffs in the number of BRs, our study also did not show differences in glycemic control when we assessed it with the latest HbA1c value.

Tildesley and cols. (8) suggested that as CSII trainers and users become more experienced in tailoring the CSII to the user, using multiple BRs may become more beneficial. However, in our sample, the patients with more BRs per day (G2) were CSII users for a longer period than the patients from G1, not corroborating the proposal by Tildesley and cols. (8).

We also found that patients with more BRs had a higher risk of hypoglycemia, reflected in the higher TBR and higher rates of asymptomatic hypoglycemia. It is important to mention that they presented a percentage of TBR above the recommended 4%-6%, as opposed to G1 (4%) (18). Considering that these patients had a greater variability of BRs and higher perfusion rates, we believe that these findings may account for the higher overall rates of hypoglycemia and asymptomatic hypoglycemia because the number of BRs may not be adjusted to patient needs/circadian rhythm. Additionally, although none of the groups had the 70% or higher TIR advocated, patients with more BRs were further from that goal, but the difference was not statistically significant (66.6% ± 15.8% vs. 59.8% ± 13.8], *P* = .789) (18).

To understand more clearly BR’s role in hypoglycemia and asymptomatic hypoglycemia, we adjusted binary logistic regression models for possible confounding factors between groups and found that the use of more than 4 BRs was an independent predictor of asymptomatic hypoglycemia in patients using CSII. Although we found disease duration, time in CSII therapy, variability of infusion rate, and basal insulin daily dose (U/kg) factors added to the regression models were not predictors of asymptomatic hypoglycemia in our sample, one could be suggest that the higher incidence of asymptomatic hypoglycemia and unawareness of hypoglycemia in patients with more than 4 BRs could be related to the longer disease duration, a known risk factor for that, but our study did not verify this (21).

We believe this is the first study relating the number of BRs with the rate of diabetes-related acute complications, and our findings are very important in clinical practices of endocrinologists, who should be aware and ask patients about these events to adjust CSII parameters more accurately. In addition, we would like to emphasize that none of the groups presented DKA episodes, so we could not evaluate this variable.

This study has some limitations related to its retrospective design. First, we could only determine statistical associations between variables. We could not establish cause-and-effect relationships. Second, this type of study is prone to more missing data, leading to the exclusion of patients, as was the case in our study (Figure 1), and the inability to assess certain relevant variables that were not available in the clinical records, namely glucose variability, severity of hypoglycemia, the time of day when the hypoglycemia event (symptomatic or asymptomatic) was most frequent, and whether there was a relationship between hypoglycemia and higher hourly basal infusion rates. We also have no data regarding catheter sites and days between catheter changes.

A prospective study assessing these variables is necessary to characterize and define more accurately the actual impact of the number of BRs on glycemic control and the prevalence of acute diabetes-related complications in patients with T1D treated with CSII.

In conclusion, it seems that programming open-loop CSII devices with more than 4 basal insulin rates does not improve metabolic control; furthermore, it represents a potential risk factor for hypoglycemia and is an independent predictor of asymptomatic hypoglycemia.

Our results also highlight that a single assessment through HbA1c is not sufficient to evaluate metabolic control and that it is important to assess other metrics, such as TBR, reported hypoglycemia, and asymptomatic hypoglycemia, for a better characterization of the glycemic profile of patients with T1D.
